# Dabigatran activates inflammation resolution by promoting fibrinogen-like protein 2 shedding and RvD5_n-3 DPA_ production

**DOI:** 10.7150/thno.50182

**Published:** 2021-02-20

**Authors:** Juan Lei, Yu Zhou, Huakan Zhao, Yu Chen, Guifang Yan, Lei Wu, Yanquan Xu, Jiangang Zhang, Xiao Zhang, Jingchun Wang, Dingshan Li, Yongsheng Li

**Affiliations:** 1 Department of Medical Oncology, Chongqing University Cancer Hospital, Chongqing 400030, China.; 2 Clinical Medicine Research Center, Xinqiao Hospital, Army Medical University, Chongqing 400037, China.

**Keywords:** fibrinogen-like protein 2, coagulation, sepsis, dabigatran, resolvin

## Abstract

**Rationale:** The interaction between coagulation and inflammation resolution remains elusive. We recently highlighted a link between fibrinogen-like protein 2 (Fgl2) and a specialized pro-resolving mediator (SPM)-n-3 docosapentaenoic acid-derived resolvin D5 (RvD5_n-3 DPA_) in sepsis. This study aimed to investigate the functions of commonly used anticoagulants warfarin, dabigatran and heparin in regulating inflammation resolution.

**Methods:** Peripheral blood was collected from clinical sepsis patients and healthy control for the determination of indicated indexes. Mouse sepsis models of zymosan-induced peritonitis and cecal ligation and puncture (CLP) were employed for the measurement of inflammation- and coagulation-related indexes. Western-blotting, ELISA and flow cytometry were applied to assess proteins. UPLC-MS/MS was used to evaluate lipid metabolites.

**Results:** Here we report that the transmembrane Fgl2 (mFgl2) was positively associated with coagulation, while soluble Fgl2 (sFgl2) level correlated with the enhanced number of peripheral blood mononuclear cells in the sepsis patients. The anticoagulants dabigatran and warfarin attenuated zymosan-induced peritonitis, which was not shared by heparin, while only dabigatran significantly improved sepsis survival in the CLP sepsis mouse model. Although these anticoagulants consistently inhibited pro-inflammatory mediators including prostaglandin E_2_ and leukotriene B_4_, only dabigatran increased sFgl2 at both the initiation and resolution phases of inflammation. Mechanistically, dabigatran elicited the shedding of sFgl2 *via* prothrombin-related metalloproteases, thereby enhanced the subsequent biosynthesis of RvD5_n-3 DPA_ *via* STAT6-ALOX15 axis. Blocking metalloproteases or ALOX15 significantly impaired dabigatran-enhanced macrophage efferocytosis *in vitro*, as well as delayed the dabigatran-accelerated inflammation resolution *in vivo*.

**Conclusions:** Our findings identify the dual anti-inflammatory and pro-resolving actions of dabigatran, through promoting sFgl2-triggered RvD5_n-3 DPA_ production, which has important implications for promoting tissue homeostasis of sepsis.

## Introduction

Timely resolution of acute inflammation is critical for homeostasis restoration 1, 2. In view of the mutual promotion of coagulation and inflammation, anticoagulation has been considered as an effective strategy for anti-inflammation 3. Three anticoagulants, with respective activities in catalyzing the inactivation of coagulation enzymes by antithrombin (heparin), decreasing vitamin K-dependent clotting factors (warfarin), and inhibiting thrombin to prevent fibrinogen conversion to fibrin (dabigatran), are most broadly used in clinics 4. A recent study showed that anticoagulants, including recombinant human activated protein C (rhAPC), antithrombin (AT), tissue factor pathway inhibitor (TFPI), unfractionated heparin (UFH), low molecular weight heparin (LMWH), and serine protease inhibitors resulted in no survival benefits in the overall sepsis population and even in the population with sepsis-induced coagulopathy 5. Of interest, dabigatran was proven efficient for preventing *Staphylococcus aureus* sepsis 6. However, whether and how coagulation regulates inflammation resolution remain largely unknown.

The activation of thrombin from prothrombin depends on prothrombinase, *i.e*. the transmembrane form of fibrinogen-like protein 2 (mFgl2), while Fgl2 protein also has a soluble cleaved form (sFgl2), which is a negative regulator of immunity 7-9. Our recent study identified that the sFgl2 ameliorates sepsis by increasing n-3 docosapentaenoic acid-derived resolvin D5 (RvD5_n-3 DPA_, also named RvDp5) 10, a specialized pro-resolving lipid mediator (SPM) derived from n-3 docosapentaenoic acid (DPA) by 15-lipoxygenase (15-LOX or ALOX15) catalyzation 11-13. Here, we report the correlation of mFgl2 and sFgl2 with coagulation and peripheral blood mononuclear cells (PBMC), as well as explore the differential roles of anticoagulants heparin, warfarin and dabigatran on inflammation resolution. Our findings uncover the dual anti-inflammatory and pro-resolving actions of dabigatran, through promoting sFgl2-triggered RvD5_n-3 DPA_ production, which has potential implications for promoting tissue homeostasis of sepsis.

## Results

### Coagulation is dysregulated in sepsis patients

**Figure 1:**
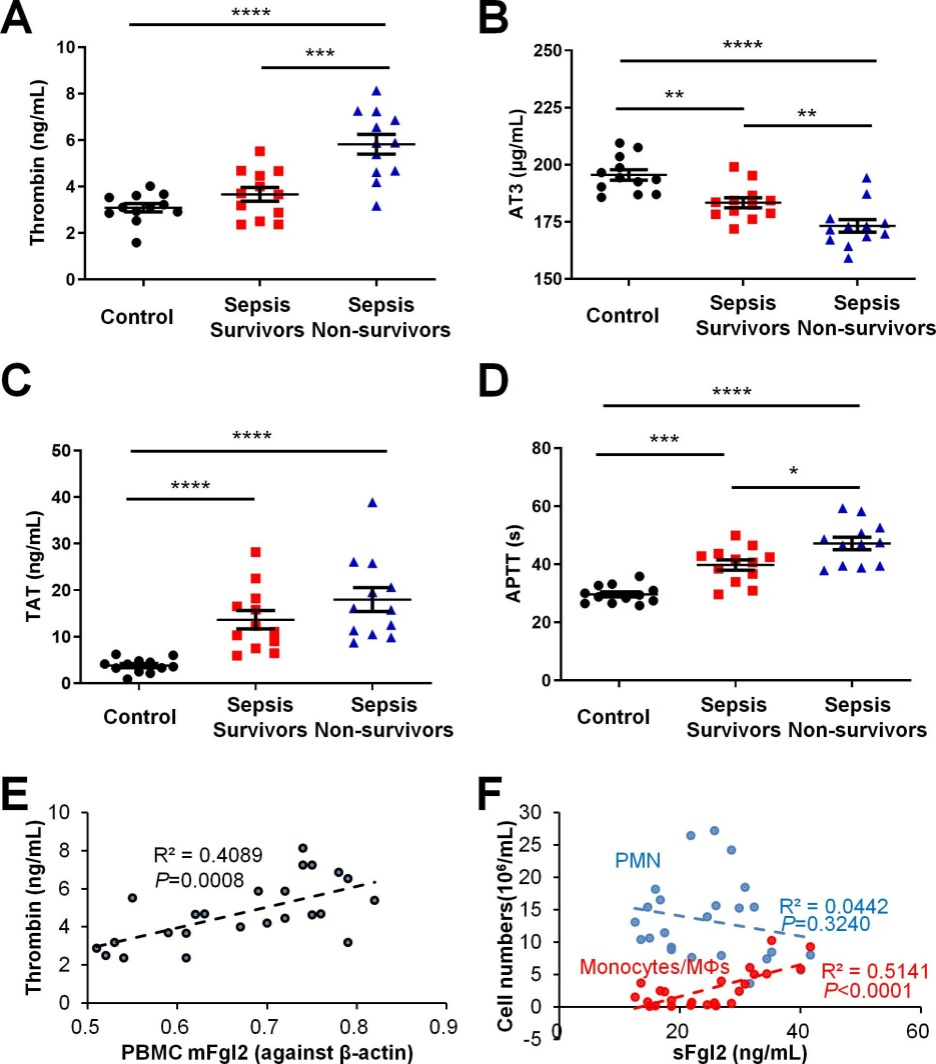
Coagulation factors in healthy control and sepsis patients. (**A**-**F**) The peripheral blood were collected from healthy control donors, sepsis survivors and non-survivors (n = 12 each) to assess levels of thrombin (**A**), AT3 (**B**), TAT (**C**) and APTT (**D**). (**E**) The correlation between PBMC mFgl2 and plasma thrombin. (**F**) The correlation between plasma sFgl2 and peripheral PMN and mononuclear cells. Error bars represent mean ± S.E.M. *P < 0.05; **P < 0.01; ***P < 0.001; ****P < 0.0001.

To evaluate the relationships between coagulation and resolution of inflammation, we assessed clotting indices in clinical samples. Sepsis patients upon Intensive Care Unit (ICU) admission within 24 h were divided into resolved (survivors) and in-hospital mortality (non-survivors). As indicated in our recent study, the sFgl2 levels were significantly lower but the mFgl2 were higher in the sepsis non-survivors *versus* survivors (see Figure 1 of ref. 10). With the increase of septic severity, here we found that the sepsis patients showed increased thrombin, thrombin-antithrombin complexes (TAT) and prolonged activated partial thromboplastin time (APTT) but reduced antithrombin III (AT3), compared with control (**Figure 1A-D**). More importantly, we found that that PBMC mFgl2 expression correlated with the blood thrombin level (**Figure 1E**). The level of sFgl2 positively correlated with the number of monocytes and macrophages, but it did not correlate with the number of polymorphonuclear neutrophils (PMN) (**Figure 1F**). Since sFgl2 is shed from mFgl2, these data are in line with our recent study, which demonstrates that sFgl2 positively correlates with inflammation resolution 10.

### Anticoagulants differentially regulates inflammation resolution

**Figure 2:**
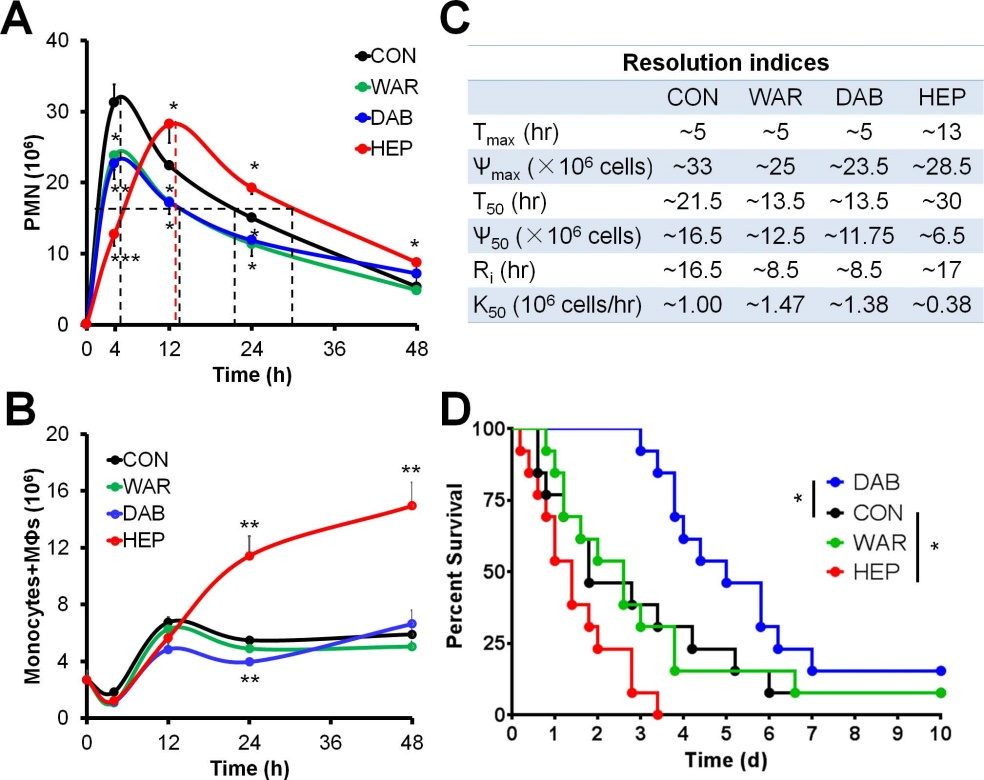
Warfarin, dabigatran and heparin differentially regulate inflammation resolution. (**A-C**) C57BL/6N mice were challenged with zymosan (Zym, 1mg *i.p.*) with PBS (control; CON), warfarin (WAR, 200 µg/kg), dabigatran (DAB, 200 µg/kg), and heparin (HEP, 200 IU/mouse). Peritoneal numbers of PMN (**A**), monocytes and macrophages (**B**) at indicated intervals were counted and resolution indices were calculated (**C**). See reference No. 10 for calculation methods of resolution indices. T_max_: time point when PMN infiltration to maximum; *Ѱ*_max_: PMN maximum number; T_50_: time point when PMN reduction to half of *Ѱ*_max_; Ѱ_50_: 50% of Ѱ_max_; *R*_i_: resolution interval, time interval from T_max_ to T_50_; *K*_50_: the rate of PMN reduction from T_max_ to T_50_. Error bars represent mean ± S.E.M. (**D**) Survival rates of CLP mice (n = 13 in each group) treated (*i.v.*) with PBS (control; CON), WAR (200 µg/kg), DAB (200 µg/kg), or HEP (200 IU/mouse). Mantel-Cox test was applied for the *P* values of survival experiments. *P < 0.05; **P < 0.01; ***P < 0.001; ****P < 0.0001 *vs.* control.

Next, the roles of three commonly used anticoagulants heparin, warfarin and dabigatran on inflammation resolution were primarily sought. The C57BL/6N mice were employed for establishing the models of zymosan-induced peritonitis and cecal ligation and puncture (CLP) (see ref. 10 for detailed methods). In the peritonitis experiments, the infiltration of peritoneal PMN of control group reached peak at ~4 hr and reduced to half at ~21.5 hr, yielding a resolution interval (*R*_i_) as ~16.5 h. Dabigatran and warfarin, but not heparin, decreased PMN infiltration into the peritoneum and accelerated the resolution of inflammation (**Figure 2A**-**B**). Of interest, heparin treatment increased the peritoneal monocytes and macrophages at the resolution phase, while dabigatran reduced the number of monocytes and macrophages at 24 hr (**Figure 2C**). In the mouse sepsis model of CLP, only dabigatran improved the survival, whereas heparin reduced survival (**Figure 2D**). Of note, the peritoneal exudates of heparin group in peritonitis and CLP models were non-clotting in every recorded time course. Together these data indicate that dabigatran, but not warfarin or heparin, was favorable for sepsis resolution.

### Dabigatran, but not heparin or warfarin, promotes sFgl2 and RvD5_n-3 DPA_ production during resolution phase in peritonitis model

**Figure 3:**
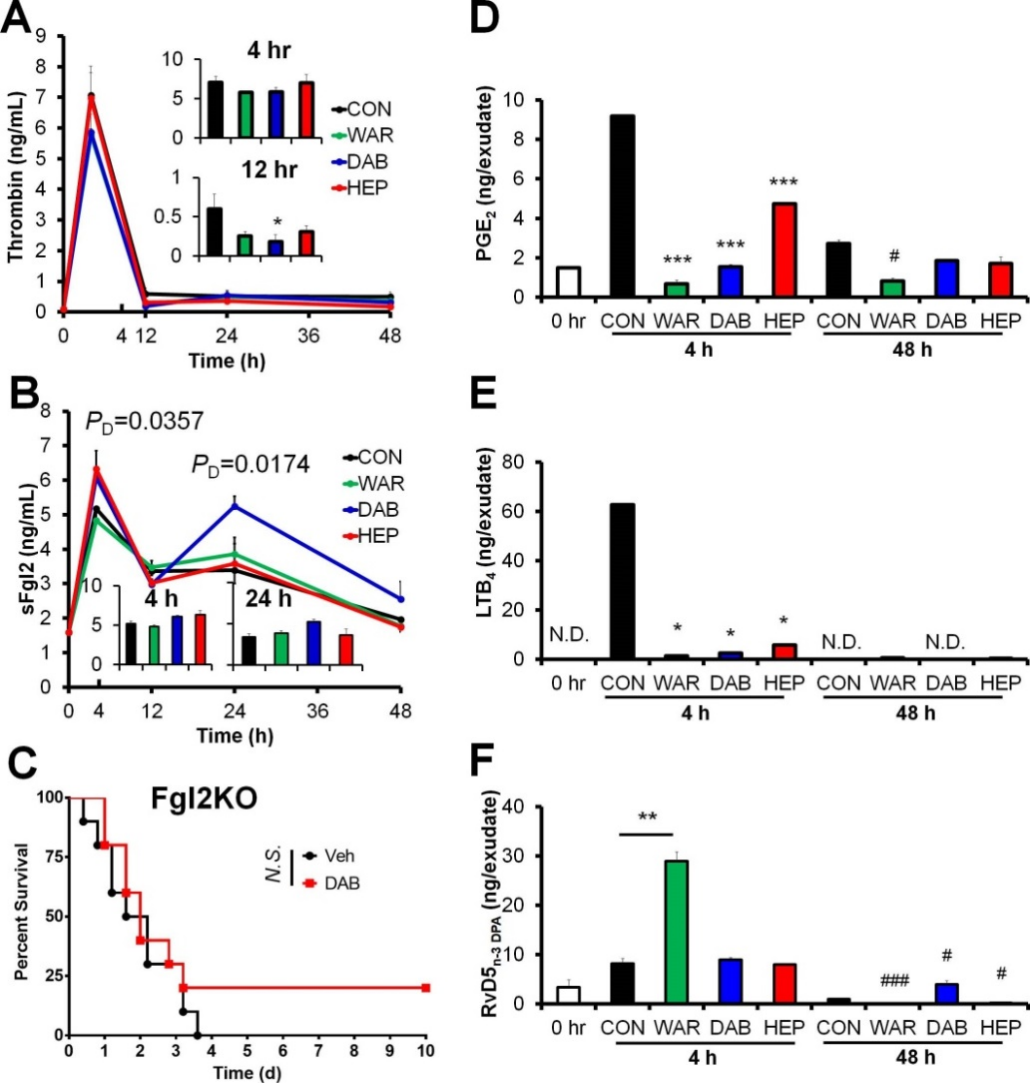
Anticoagulants differentially regulate coagulation factors and eicosanoids. (**A** and** B**) C57BL/6N mice were challenged with zymosan (Zym, 1mg *i.p.*) plus N.S. (0.9% Natrii Chloride) (CON), warfarin (WAR, 200 µg/kg), dabigatran (DAB, 200 µg/kg), or heparin (HEP, 200 IU/mouse). The peritoneal exudate thrombin (**A**) and sFgl2 (**B**) at indicated intervals were assessed with ELISA. Error bars of represent mean ± S.E.M. *P*_D_ value represents CON vs. DAB. *P < 0.05 *vs.* control. (**C**) Survival rates of CLP Fgl2KO mice (n = 10 each group) after treatment with N.S. or dabigatran (DAB, 200 µg/kg). Mantel-Cox test was applied for the *P* values of survival experiments. (**D-F**) After mice were treated as indicated in (A and B), peritoneal exudate samples at 4 and 48 h were collected to assess the levels of PGE_2_ (**D**), LTB_4_ (**E**) and RvD5_n-3 DPA_ (**F**) at indicated intervals with UPLC-MS/MS. ND denotes non-detected. Error bars of represent mean ± S.E.M. *P < 0.05; **P < 0.01; ***P < 0.001 *vs.* 4hr control. ^#^*P* < 0.05 and ^###^*P* < 0.001 *vs*. 48 hr control.

Since dabigatran is an inhibitor of thrombin 4, the above results inspired us to determine thrombin and sFgl2 to validate whether dabigatran enhanced sFgl2-related inflammation resolution. Among these anticoagulants, only dabigatran significantly inhibited thrombin at 12 hr post zymosan injection (**Figure 3A**), and increased sFgl2 production at both 4 and 48 hr (**Figure 3B**). Our previous study showed that Fgl2 deficiency delayed inflammation resolution 10, here we found that dabigatran did not improve the sepsis survival in Fgl2 knockout mice (**Figure 3C**), indicating that Fgl2 was required for dabigatran-improved sepsis resolution.

Using UPLC-MS/MS, we performed metabololipidomics with exudates from zymosan-induced peritonitis model. The lipid mediators derived from docosahexaenoic acid (DHA), eicosapentaenoic acid (EPA), docosapentaenoic acid (DPA) and arachidonic acid (AA) were differentially regulated by heparin, warfarin and dabigatran (Table S1). As expected, all three anticoagulants reduced the pro-inflammatory eicosanoids prostaglandin E_2_ (PGE_2_) and leukotriene B_4_ (LTB_4_) in mouse peritoneal exudates (**Figure 3D-E**), delineating the anti-inflammatory actions of anticoagulants. Our previous study indicated that sFgl2 promoted inflammation-resolution by enhancing the biosynthesis of RvD5_n-3 DPA_ 10. Herein, although RvD5_n-3 DPA_ was upregulated by warfarin in the early phase of the challenge, this increase was not sustained into the resolution phase (**Figure 3F**). Distinct from warfarin and heparin, dabigatran boosted RvD5_n-3 DPA_ production during the resolution interval (**Figure 3F**). These data demonstrate that dabigatran promotes the production of sFgl2 and RvD5_n-3 DPA_ during inflammation resolution.

### Dabigatran elicits RvD5_n-3 DPA_ production dependent on ADAMs-mediated sFgl2 shedding

**Figure 4:**
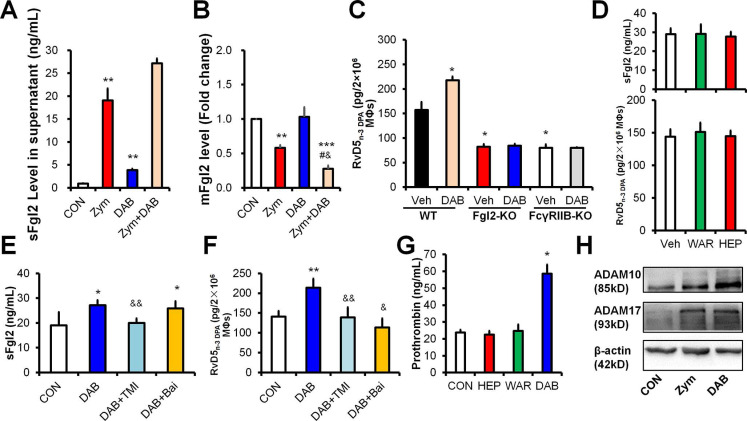
Dabigatran promotes sFgl2 shedding to increase RvD5_n-3 DPA_ production. (**A** and **B**) C57BL/6N murine peritoneal macrophages (MΦs) were treated with PBS (control; CON), zymosan (Zym, 100 ng/mL) or dabigatran (DAB, 100 ng/mL) for 4 h, the levels of sFgl2 (**A**) and mFgl2 (**B**) were determined with ELISA. (**C**) Peritoneal MΦs from WT, Fgl2-KO and FcγRIIB-KO mice were treated with/without DAB (100 ng/mL) for 4 h, the supernatant RvD5_n-3 DPA_ were determined with UPLC-MS/MS. (**D**) Mouse peritoneal MΦs were treated with PBS (control; CON), warfarin (WAR, 100 ng/mL) or heparin (HEP, 3 U/mL) for 4 h, the supernatant sFgl2 and RvD5_n-3 DPA_ were determined with ELISA and UPLC-MS/MS, respectively (n = 3); ns denotes no significant difference. (**E** and **F**) Murine peritoneal macrophages were treated with DAB (100ng/mL), TMI-1 (10 µM) and baicalein (Bai, 10 µM) for 4 h, the secretion of sFgl2 (**E**) and RvD5_n-3 DPA_ (**F**) were determined with ELISA and UPLC-MS/MS, respectively. (**G**) Mouse peritoneal macrophages were treated with/without PBS (control), HEP (3 U/mL), WAR (100 ng/mL) or DAB (100 ng/mL) for 4 h, the supernatant prothrombin were determined with ELISA. (**H**) Mouse peritoneal MΦs were treated with/without Zym (100 ng/mL) or DAB (100 ng/mL) for 24 h, ADAM10 and ADAM17 were determined with Western-blotting. Veh denotes vehicle. Error bars represent mean ± S.E.M. *P < 0.05 and **P < 0.01 *vs.* control or WT vehicle. ^#^*P* < 0.05 *vs*. Zymosan. ^&^*P* < 0.05 and ^&&^*P* < 0.01* vs.* dabigatran.

To address the underlying mechanism, we treated mouse peritoneal MΦs with zymosan and dabigatran, respectively. Zymosan increased sFgl2 and decreased mFgl2, and these actions were further enhanced by dabigatran in MΦs (**Figure 4A-B**). Of interest, dabigatran failed to increase RvD5_n-3 DPA_ in MΦs with the deficiency of Fgl2 or its receptor FcγRIIB (**Figure 4C**), indicating that dabigatran increased RvD5_n-3 DPA_ *via* the sFgl2-FcγRIIB pathway. However, heparin and warfarin did not share the function of dabigatran on the production of sFgl2 and RvD5_n-3 DPA_ (**Figure 4D**).

We previously reported that sFgl2 was produced by metalloproteases (ADAM10 and ADAM17)-dependent ectodomain shedding.^10^ Here we show that dabigatran-enhanced production of sFgl2 and RvD5_n-3 DPA_ could be reversed by both ADAMs inhibitor TMI-1 and lipoxygenases inhibitor baicalein in macrophages* in vitro* (**Figure 4E-F**). Since the prodomain of ADAMs contains a consensus FXa cleavage site (I-E/D-G-R̂) conserved in factor II (prothrombin) 14, we wondered whether prothrombin could promote ADAMs-mediated sFgl2 shedding. We found that prothrombin was upregulated by dabigatran, but not by heparin or warfarin *in vivo* (**Figure 4G**). Moreover, similar to zymosan, dabigatran boosted both ADAM10 and ADAM17 expression in MΦs (**Figure 4H**). Given that dabigatran increased sFgl2 secretion but decreased mFgl2 in macrophages (**Figure 4A-B**), these results suggest that dabigatran may increase ADAMs *via* prothrombin, thereby feedback promoting sFgl2 shedding and subsequent RvD5_n-3 DPA_ production.

### Dabigatran promotes RvD5_n-3 DPA_ production *via* sFgl2-STAT6-ALOX15 pathway

**Figure 5:**
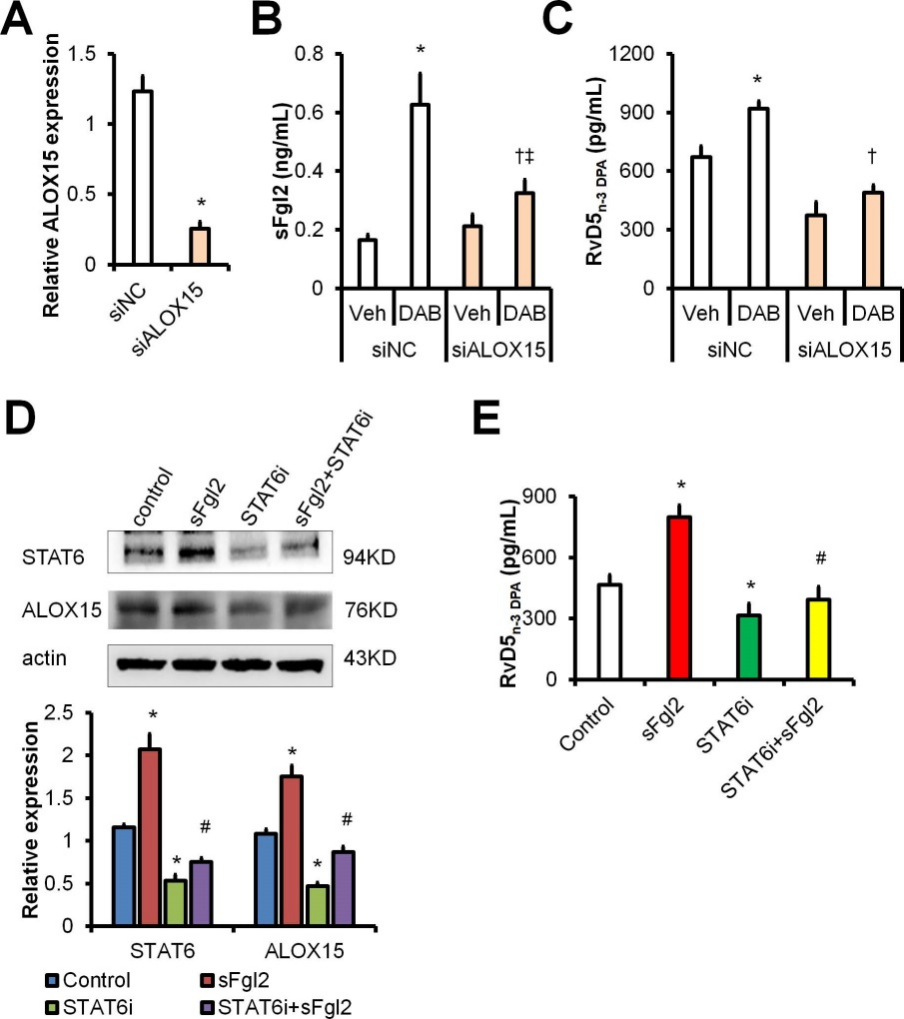
Dabigatran promotes RvD5_n-3 DPA_ production via sFgl2-STAT6-ALOX15 pathway. **(A-C)** BMDMs were transfected with siNC or siALOX15 for 48 h. (**A**) *ALOX15* mRNA expression levels were assessed with qPCR. BMDMs were treated with PBS or dabigatran (100ng/mL) for 4 h, the supernatant sFgl2 (**B**) and RvD5_n-3 DPA_ (**C**) were determined with ELISA. (**D**) BMDMs were treated with sFgl2 (1 µg/mL) and/or STAT6i (AS1517499, 100 nM) for 30 min, the expression of STAT6 and ALOX5 were assessed with Western-blotting. (**E**) BMDMs were treated with sFgl2 (1 µg/mL) and/or STAT6i (AS1517499, 100 nM) for 4 h, the supernatant RvD5_n-3 DPA_ was detected with UPLC-MS/MS. Error bars represent mean ± S.E.M. *P < 0.05* vs*. siNC vehicle or control;^ †^*P* < 0.05* vs*. siNC *plus* DAB; ^‡^*P* < 0.05* vs*. siALOX15 vehicle; ^#^*P* < 0.05* vs*. sFgl2.

We identified earlier that sFgl2 initiates ALOX15 expression to enhance RvD5_n-3 DPA_ biosynthesis during inflammation resolution 10. To further address how dabigatran facilitates RvD5_n-3 DPA_ production, we performed RNA interference against ALOX15 in MΦs (**Figure 5A**) and found that dabigatran-induced production of sFgl2 and RvD5_n-3 DPA_ were dampened by ALOX15 knockdown (**Figure 5B-C**).

It has been reported that STAT6 is involved in ALOX15 transcription 15, we wondered whether sFgl2 upregulated ALOX15 *via* STAT6 pathway. We found that STAT6 inhibitor (STAT6i) AS1517499 blocked both expressions of STAT6 and ALOX15 (**Figure 5D**). Moreover, STAT6i blunted sFgl2-initated RvD5_n-3 DPA_ production (**Figure 5E**). Owing to that dabigatran treatment increased RvD5_n-3 DPA_ through activating sFgl2 shedding, these data indicate that dabigatran induces RvD5_n-3 DPA_ biosynthesis* via* sFgl2-STAT6-ALOX15 signaling pathway.

### ADAMs and lipoxygenases are required for dabigatran-accelerated resolution of inflammation

**Figure 6:**
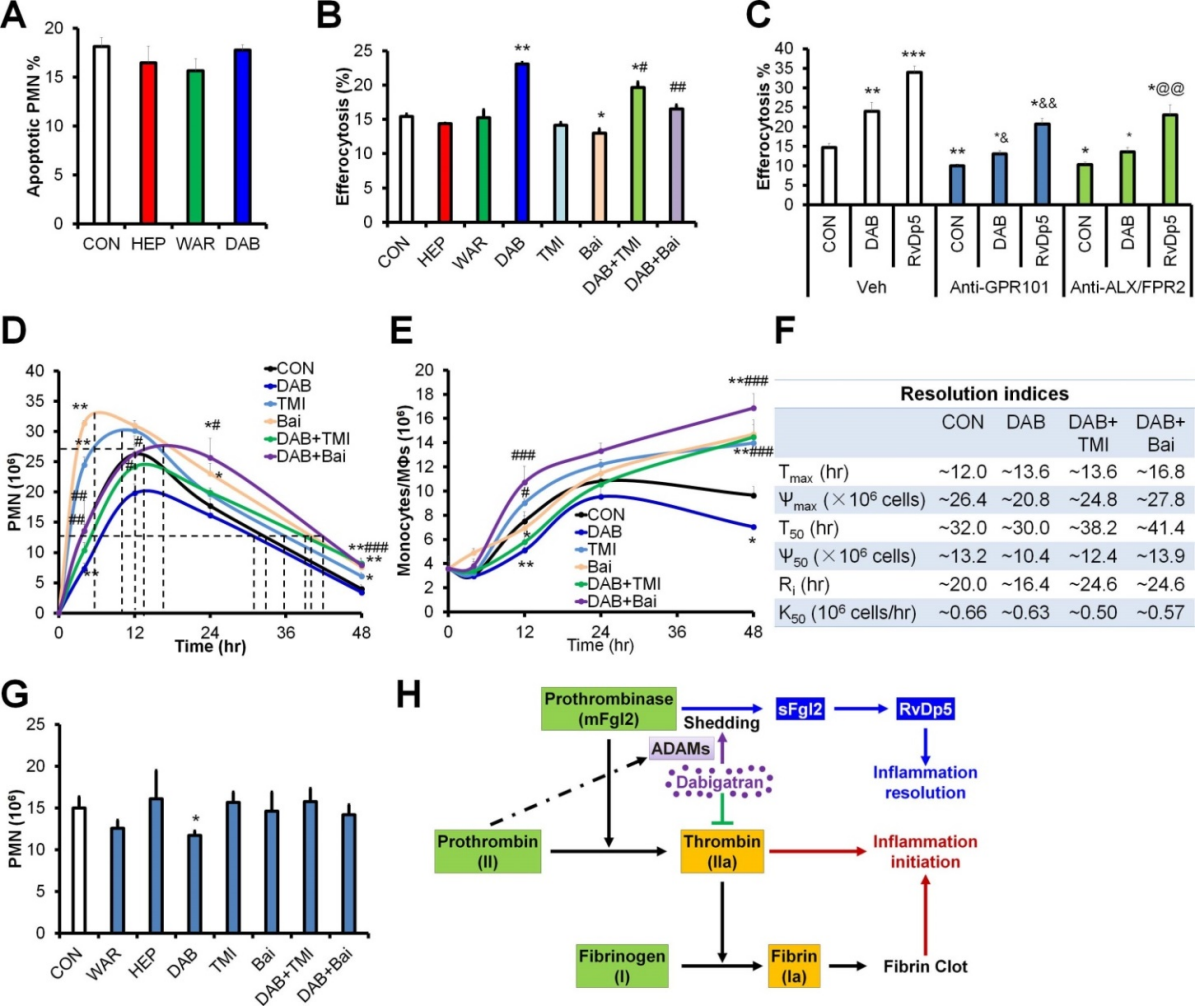
Dabigatran promotes inflammation resolution dependent on ADAMs and ALOX15. (**A**) Mouse PMN was treated with PBS (control; CON), heparin (HEP, 3 U/mL), warfarin (WAR, 100 ng/mL) and dabigatran (DAB, 100 ng/mL) for 6 h, the apoptotic PMN was determined with flow cytometry. (**B**) After mouse BMDMs were pretreated with PBS (control; CON), heparin (HEP, 3 U/mL), warfarin (WAR, 100 ng/mL), dabigatran (DAB, 100 ng/mL), TMI-1 (10 µM), and baicalein (Bai, 10 µM) for 4 h, they were co-cultured with CFSE-labeled apoptotic MC38 cells at the ratio of 1:10 for 30 min, efferocytosis was performed with flow cytometry. (**C**) After mouse BMDMs were pretreated with PBS (control; CON), dabigatran (DAB, 100 ng/mL), or RvD5_n-3 DPA_ (10 nM) with or without Anti-GPR101 (1:100) or anti-ALX/FPR2 (1:100) for 4 h, then they were co-cultured with CFSE-labeled apoptotic MC38 cells at the ratio of 1:10 for 30 min, efferocytosis was performed with flow cytometry. *P < 0.05 and **P < 0.01 vs. control. ^#^*P* <0.05; ^##^*P* < 0.01 and ^###^*P* < 0.001 vs. dabigatran. (**D-F**) C57BL/6J mice were challenged with zymosan (Zym, 1mg *i.p.*) and treated with PBS (control; CON), dabigatran (DAB, 200 µg/kg), TMI-1 (200 µg/kg) and baicalein (Bai, 10 mg/kg). Peritoneal numbers of PMN (**D**), monocytes and macrophages (**E**) at indicated intervals were counted and resolution indices were calculated (**F**). (**G**) C57BL/6J mice were challenged with zymosan (Zym, 1mg *i.p.*), at 12 hr post injection, PBS (control; CON), warfarin (WAR, 200 µg/kg), dabigatran (DAB, 200 µg/kg), heparin (HEP, 200 IU/mouse), TMI-1 (200 µg/kg) and baicalein (Bai, 10 mg/kg) were administrated i.p., the peritoneal exudates were collected for counting PMN numbers. Error bars represent mean ± S.E.M. *P < 0.05 and **P < 0.01 *vs.* control. ^#^*P* < 0.05; ^##^*P* < 0.01 and ^###^*P* < 0.001 *vs*. dabigatran; ^&^*P* < 0.05 and ^&&^*P* < 0.01 *vs.* Anti-GPR101 control; ^@@^*P* < 0.01 *vs.* Anti-ALX/FPR2 control. (**H**) Schematic of dabigatran dual function in inflammation resolution.

To further explore the role of anticoagulants on inflammation resolution, we assessed PMN apoptosis and macrophage efferocytosis *in vitro*. We found that all three anticoagulants did not change the PMN apoptosis, while dabigatran significantly promoted apoptotic cell efferocytosis by macrophages (**Figure 6A-B**). Inhibition of ADAMs (by TMI-1) alone did not significantly altered macrophage efferocytosis, while baicalein (lipoxygenases inhibitor) alone inhibited efferocytosis. Of note, both TMI-1 and baicalein impaired the dabigatran-upregulated efferocytosis (**Figure 6B**), suggesting that dabigatran enhances macrophage efferocytosis *via* ADAMs and lipoxygenases.

It has been reported that RvD5_n-3 DPA_ display pro-resolving actions by activating G-protein coupled receptors GPR101 and ALX/FPR2 10, 16. To address whether dabigatran promoted inflammation resolution *via* RvD5_n-3 DPA_, we determined efferocytosis of macrophages after pre-treated with antibodies against GPR101 and ALX/FPR2. We found that both anti-GPR101 and anti-ALX/FPR2 blocked the efferocytosis of macrophages induced by dabigatran and RvD5_n-3 DPA_ (**Figure 6C**), indicating that RvD5_n-3 DPA_ and its receptors were required for dabigatran induced efferocytosis.

Since the macrophage efferocytosis is essential for apoptotic PMN clearance and homeostasis restoration during inflammation resolution, we established the peritonitis model with the C57BL/6J mice, another mouse substrain and monitored the peritoneal infiltrated PMN and macrophages *in vivo*. Distinct from the C57BL/6N mice, the time to the maximum PMN infiltration was delayed to ~12 hr in C57BL/6J mouse model (**Figure 6D-F**), which is consistent with previous studies that C57BL/6J and C57BL/6N mice are differentially susceptible to inflammation stimulation 17, 18. Dabigatran inhibited peritoneal PMN infiltration and accelerated PMN reduction during the resolution phase, which was reversed by TMI-1 and baicalein (**Figure 6D**). Both of TMI-1 and baicalein alone promoted PMN infiltration during the initiation phase of inflammation and delayed resolution of peritonitis (**Figure 6D** and** 6F**). Of note, the number of peritoneal monocytes and macrophages was decreased by dabigatran treatment, which was impaired by TMI-1 or baicalein (**Figure 6E**). We next administrated these treatments at the peak of inflammation (*i.e*. 12 hr post zymosan injection) and assessed PMN numbers at 24 hr. Only dabigatran significantly reduced PMN infiltration, which could be reversed by TMI-1 or baicalein (**Figure 6G**). These results identify the dual anti-inflammatory and pro-resolving actions of dabigatran (**Figure 6H**).

## Discussion

Coagulation promotes the initiation of multiple types of inflammation, thus anticoagulation has been considered as an effective anti-inflammatory strategy 3. However, the role of coagulation on inflammation resolution was unknown. In the present study, we found that sFgl2, the prothrombinase mFgl2 cleaved product, correlated with inflammation resolution. We compared the differential roles of three common clinical used anticoagulants heparin, warfarin and dabigatran in mice models of CLP and yeast wall zymosan-induced peritonitis. Our data showed that only dabigatran significantly improved sepsis outcome. Mechanistically, dabigatran inhibited thrombin, increased prothrombin-related ADAMs which promoted sFgl2 shedding, thereby subsequently increased RvD5_n-3 DPA_ that accelerated inflammation resolution.

As mentioned above, samples from heparin group showed non-clotting in every recorded time course of peritonitis and CLP model. Heparin displays multiple functions: it activates AT3 which binds to thrombin (IIa), and factor II (prothrombin), VII, IX and X to block the coagulation activity of these molecules 19; promotes the binding of thrombin to fibrin polymer 20; binds to platelet factor 4 (PF4) and results in heparin-induced thrombocytopenia (HIT) 21; increases the permeability of blood vessel walls by promoting bradykinin formation 22. Heparin has known anti-inflammatory effects, both described in multiple experimental studies and clinically 23. In our present study, we also found that heparin was able to inhibit pro-inflammatory LTB_4_ and PGE_2_. Of interest, the PMN infiltration into the peritoneum was delayed by heparin, whereas the resolution was also prolonged. It also significantly increased the monocytes and macrophages during the resolution phase. However, heparin could not enhance macrophage efferocytosis *in vitro*. These results suggested that heparin is not recommended for use in patients with sepsis, because it delays sepsis resolution and deteriorates the outcome.

It is well known that the vitamin K-dependent warfarin takes at least 3-5 days to reduce the synthesis of coagulation factors II, VII, IX and X in the liver. However, warfarin was also reported to inhibit leukocyte migration in acute inflammatory exudates 24. In our experiments, unlike dabigatran, warfarin increased peritoneal exudates RvD5_n-3 DPA_ at the initial stage of inflammation. Warfarin also inhibited LTB_4_ and PGE_2_ production, delineating its anti-inflammatory action in acute inflammation. However, it didn't promote sFgl2 secretion in peritoneal exudates and RvD5_n-3 DPA_ production in MΦs. It is known that the carboxylation in prothrombin synthesis is linked to the metabolism of the vitamin-specifically the cyclic interconversion of vitamin K and vitamin K epoxide. Actually, the primary site of action of WAR appears to be an inhibition of the epoxide-to-vitamin K conversion 25. Of interest, the redox pathways also contribute to the production of both pro-inflammatory lipids (*e.g.* LTB_4_ and PGE_2_) and SPMs (*e.g.* resolvins and protectins) 11, 26, and our present data showed that the production of LTB_4_ and PGE_2_ were inhibited by warfarin. Although RvD5_n-3 DPA_ was upregulated by warfarin in the early phase of the challenge, this increase was not sustained into the resolution phase. Hence the underlying anti-inflammatory mechanism during initiation stage may be related to the inhibition of redox pathways regulated by the epoxy reductase and unrelated to vitamin K-dependent coagulation factor synthesis, which awaits further investigation.

Dabigatran is a non-vitamin‐K anticoagulant. Its classical anticoagulant mechanism is directly inhibiting the active center of thrombin, preventing fibrinogen from cleavage into fibrin, thereby blocking the clotting cascade and thrombosis. We showed that dabigatran increased sFgl2 but decreased mFgl2 and thrombin. Moreover, prothrombin was upregulated after dabigatran administration, but neither by heparin nor by warfarin. As well-known, prothrombin is converted to thrombin *via* the catalyzation of prothrombinase, dabigatran can directly inhibit prothrombinase, thus the conversion reaction is blunted and prothrombin is upregulated by dabigatran. Since ADAMs cleave prothrombin at the consensus FXa cleavage site (I-E/D-G-R̂) 14, dabigatran may promote ADAMs expression *via* prothrombin, thereby increasing the shedding of sFgl2. Of note, the life-threatening effects of dabigatran has also been reported in some case reports 27, 28. Moreover, Essentials Reversal of anticoagulant effects of dabigatran and increased gastrointestinal bleeding may occur, monitoring of its level after antidote application is crucial to detect rebound 29. Therefore, the dosage and mode of administration of dabigatran in different diseases and stages need to be further explored. Furthermore, the roles of downstream molecules of thrombin, including fibrinogen, protease activated receptors, and protein C in inflammation resolution were not addressed in our present study. Nonetheless, Dabigatran has the advantages that it can be taken orally, fewer major bleeding complications, in particular intracranial hemorrhage, and has fewer drug interactions.

Together our results demonstrate that warfarin, heparin and dabigatran play differential roles on inflammation resolution. Dabigatran can be recommended as a protective drug for sepsis since it displays dual anti-inflammatory and pro-resolving actions.

## Materials and methods

### Human and mice samples

The characteristics for the clinical samples were indicated in the Table S1 of our recent study 10. Male C57BL/6 mice were purchased from the Animal Institute of the Academy of Medical Science (Beijing, China) and were kept under specific pathogen-free conditions. Fgl2KO and FcγRIIBKO mice were respectively provided by Dr. Steve Smiley (The Trudeau Institute, NY, USA) and Dr. J. Sjef Verbeek (Leiden University Medical Center, the Netherlands). Mice *in vivo* experiment protocols including zymosan-induced peritonitis and CLP were described previously (see ref 10, 26 for details). All human and animal experiments meet the ethical principles and requirements of the committees of the Army Medical University and Wenzhou Medical University, and complies with the Declaration of Helsinki, as well as the IRB, IACUC and/or ARRIVE 30 guidelines. After screening with normal distribution, we selected samples that match conditions for statistical analysis. Warfarin (Cat No. HY-B0687), dabigatran (Cat No. HY-10163), and heparin (Cat No. HY-17567) were purchased from MedChemexpress (Princeton, NJ). We normalized the samples before statistics, and selected effective concentrations of compounds after preliminary experiments.

### Macrophage isolation

MΦs were isolated from the peritoneal cavity of C57BL/6 mice by flushing the peritoneal cavity with 5 mL ice cold PBS. Cells were then centrifuged and resuspended in serum free RPMI-1640 (Gibco) and plated with a density of 20000 cells/cm^2^ and incubated at 37 °C and 5% CO_2_ for 1-2 hours. Subsequently, non-adherent cells were washed away with PBS. The majority remaining cells were MΦs which were incubated in RPMI with 10% fetal bovine serum (FBS, Gibco) over night before stimulation.

For the preparation of bone marrow derived macrophages (BMDMs), single-cell suspensions of bone marrow (BM) cells were collected from 8-10 week-old mice with PBS flushing. Cells were differentiated into MΦs by incubation in complete RPMI-1640 supplemented with 10% FBS (Gibco) and 20 ng/mL granulocyte-macrophage colony stimulating factor (GM-CSF, Cat No. 315-03, PeproTech) for 7 days.

### Western-blotting and ELISA

Western-blotting and ELISA was performed as described previously 10. The STAT6 inhibitor AS1517499 was purchased from Selleck (Cat No. S8685). The primary antibodies STAT6 (AF8068) was from Beyotime (Wuhan, China), ALOX15 (H00000246-D01P) was bought from Novus Biologicals (Centennial, CO). The primary antibodies ADAM10 (Cat No. AB19026) and ADAM17 (Cat No. AB19027) were bought from Merck Millipore (Darmstadt, Germany) and were incubated with the PVDF membranes containing respective target proteins according to the manufacturer's instructions. ELISA kits for human thrombin (Cat No. ab108909), mouse thrombin (Cat No. ab230933) and prothrombin (Cat No. ab157526) were obtained from Abcam (Cambridge, MA), for mouse sFgl2 (Cat No. 437808) was from BioLegend (Dedham, MA). For the experiments of mFgl2 determination, cell proteins were collected with a Membrane and Cytoplasmic Protein Extraction kit (Cat No. C510005, Sangon Biotech, Shanghai, China) and assessed with ELISA (Cat No. 436907 for human and catalog no. 437808 for mouse; BioLegend) and were normalized with b-actin (PathScan Total b-Actin Sandwich ELISA Kit, Cat No. 7880, Cell Signaling Technology).

### Real-time quantitative PCR (qPCR)

RNA were extracted by RNAiso Plus (Cat No. 9109, TaKaRa, Otsu, Shiga, Japan) according to the manufacturer's instructions, and then were reversely transcribed into cDNA using Takara PrimeScript RT reagent Kit with gDNA Eraser. The expression of ALOX15 was determined with qPCR which was performed as indicated previously 10. qPCR was performed using a 7900HT Fast Real-Time PCR system (Applied Biosystems). The mRNA expression levels were normalized to β-ACTIN. The primers are as follows: ALOX15-F GGCTCCAACAACGAGGTCTAC and ALOX15-R CCCAAGGTATTCTGACACATCC; β-ACTIN-F TGACAGGATGCAGAAGGAGA and β-ACTIN-R GTACTTGCGCTCAGGAGGAG.

### Flow cytometry

Cells were blocked with anti-CD16/CD32 (BD) to block nonspecific binding, followed by surface staining with CD11b (clone M1/70, Cat No. 101206, Biolegend, San Diego, CA), F4/80 (clone BM8, Cat No. 123110 and 123116, Biolegend), Ly6G (clone 1A8, Cat No. 127618, Biolegend), Annexin V (Cat No. 640930, Biolegend) and mFgl2 (clone 6D9, Cat No. H00010875-M01, Novus) all diluted in PBS without Ca^2+^/Mg^2+^ supplemented with 2% FCS and 2 mM EDTA. Samples were then acquired on FACS Canto II (BD) and data was analyzed with flowjoV10 (Tree Star, Inc., Ashland, OR).

### UPLC-MS/MS

The metabololipidomics was performed using a UPLC I-Class system (Waters, Milford, MA, USA) equipped with an AB Sciex Instruments 6500 Q-TRAP mass spectrometer (Applied Biosystems, Foster City, CA, USA) as described previously 10. Prior to sample extraction, deuterated internal standards d8-5S-HETE, d4-PGE_2_, d4-LTB_4_, d5-LXA_4_, and d5-RvD2 (500 pg each) were added to facilitate quantification. The lipid metabolites were isolated by solid phase extraction on a C18 column (6 mL, 500 mg, 37~55 μm particle, Waters). Samples were washed with 10 mL of water and 6 mL of n-hexane, dried and eluted by gravity with 8 mL of methyl formate. Extracted samples were separated by the Acquity UPLC system. The column (Acquity UPLC BEH C18 , 2.1 × 100 mm; 1.7 μm; Waters) was eluted at a flow rate of 0.2 mL/min with MeOH/water/acetic acid (60/40/0.01, v/v/v) ramped to 80/20/0.01 (v/v/v) after 5 min, 95/5/0.01 (v/v/v) after 8 min and then to 100/0/0.01 (v/v/v) for the next 4 min, subsequently returned to 60/40/0.01(v/v/v) and maintained for 5 min. The column temperature was kept at 40 °C and a 10 μL aliquot of each sample was injected onto the column. Mass spectrometry was performed on the AB Sciex 6500 QTRAP, triple quadrupole, linear ion trap mass spectrometer equipped with a Turbo V ion source. Lipid mediators (LMs) were detected in negative electrospray ion (ESI) mode. Nitrogen was employed as the collision gas. Curtain gas (CUR), nebulizer gas (GS1), and turbo-gas (GS2) were set at 10 psi, 30 psi, and 30 psi, respectively. The electrospray voltage was -4.5 kV, and the turboion spray source temperature was 550 °C. LMs were analyzed using multiple reaction monitoring (MRM). Mass spectrometer parameters including the declustering potentials and collision energies were optimized for each analyze. Dwell time was also tuned individually and minimum data points of peaks were set as 4 according to the guidance of instruments. All samples were kept at 4 °C throughout the analysis and detected in triplicate. Data acquisitions and analysis were performed using Analyst 1.6.2 and Multiquant V1 software (Applied Biosystems). The retention time of each LM was validated by authentic standards with maximum drift as 0.05 s. A minimum of 6 diagnostic ions and retention time were used for lipid identification. The limits of detection (LOD) and quantification (LOQ) were determined at a concentration where the S/N ratios were 3 and 10, respectively. The peak area of MRM transitions with a minimum area of 2000 counts and linear calibration curve (10, 30, 50, 100, 200, 500, 800, 1000 pg with an r^2^ values of 0.98-0.99) of each compound were used to quantify the compounds.

### Efferocytosis

Mouse MC38 cells were seeded at 1 × 10^6^ cells/mL onto 35-mm plates, irradiated with UV-C light (254 nm) for 20 minutes, and incubated at 37 °C for 2 hours. Apoptotic (annexin V-positive and annexin V/PI-double-positive) cells were verified by flow cytometry using the APC Annexin V Apoptosis Detection Kit with PI (BioLegend). These were washed with PBS and labeled with 1 μM CFSE (5(6)-carboxyfluorecein diacetate succinimidyl ester, MCE, Cat. No. HY-D0938) in PBS for 30 minutes at room temperature. Bone marrow-derived macrophages were seeded onto 96-well plates at 4 × 10^4^ cells/well and were stained with F4/80 (Biolegend) for 20 min at 37 °C to visualize cell membranes, washed with RPMI-1640, and incubated with RvD5_n-3 DPA_ (10 nM), DAB (100 ng/mL), warfarin (100 ng/mL), heparin (HEP, 3 U/mL) or control (PBS) for 30 minutes at 37 °C. Apoptotic CFSE-labeled MC38 cells were added directly to the macrophages at a 10:1 ratio (apoptotic MC38/macrophage), and the increase in CFDA^+^F4/80^+^ percentage was quantified using flow cytometry.

For the preincubation experiments of antibodies, the anti-GPR101 antibody (clone G278, host: rabbit, Assay Biotechnology) or anti-ALX mAb (10 μg/mL, clone FN-1D6-A1, Genovac, Freiburg, Germany), or isotype control mouse IgG1 (BD biosciences) was incubated with cells on microtiter plates at 1:100 dilutions for 20 minutes before addition of the indicated mediators.

### Statistical analysis

Statistical analysis was performed with Excel 2010 (Microsoft) and Prism 7 software (GraphPad Software). Results are expressed as mean ± SEM. All representative experimental findings were verified in at least three independent experiments. Student's *t*-test and ANOVA were used to compare differences between groups. Mantel-Cox test was applied to compare survival rates between groups. *P* < 0.05 was considered statistically significant.

## Supplementary Material

Supplementary table S1.

## Abbreviations

ADAM: ADAM metallopeptidase
APTT: prolonged activated partial thromboplastin time
AT: antithrombin
CLP: cecal ligation and puncture
DPA: docosapentaenoic acid
ICU: Intensive Care Unit
LMWH: low molecular weight heparin
LOX: lipoxygenase
LTB_4_: leukotriene B_4_
mFgl2: transmembrane fibrinogen-like protein 2, prothrombinase
MRM: multiple reaction monitoring
PGE_2_: prostaglandin E_2_
rhAPC: recombinant human activated protein C
*R*_i_: resolution interval
RvD5_n-3 DPA_ or RvDp5: n-3 docosapentaenoic acid-derived resolvin D5
sFgl2: soluble fibrinogen-like protein 2
SPM: specialized pro-resolving lipid mediator
TAT: thrombin-antithrombin complexes
TFPI: tissue factor pathway inhibitor
UFH: unfractionated heparin
UPLC-MS/MS: ultra-performance liquid chromatography tandem mass spectroscopy
