# Supplementary material for: Dabigatran activates inflammation resolution by promoting fibrinogen-like protein 2 shedding and RvD5n-3 DPA production
Source: Theranostics. 2021 Feb 20;11(9):4251–61. doi: 10.7150/thno.50182 (PMC7977467; doi:10.7150/thno.50182)
Supplement: Supplementary file 1 — Supplementary table S1. [file thnov11p4251s1.pdf]

Table S1. Lipidome in exudates from zymosan-initiated peritonitis treated with N.S. (0.9% Natrii Chloride) (CON), warfarin (WAR, 200 μg/kg), dabigatran (DAB, 200 μg/kg), or heparin (HEP, 200 IU/mouse) for 4 and 48 hrs. Data are shown as mean ± SD (pg/exudate). ND, non-detected.

**Lipid Mediators (pg/exudate)**	0 hr	4 hr				48 hr			
		CON	WAR	DAB	HEP	CON	WAR	DAB	HEP
AA Metabolome									
AA	317±324	4210±1057	2440±743	4048±4936	20405±33552	76±40	285±323	364±594	97±74
PGD_2	2.17±2.06	3.76±1.26	3.02±0.46	2.45±1.18	126.83±226.45	1.73±0.97	0.73±0.45	1.73±0.85	2.06±1.67
PGE_2	1.48±0.21	9.17±1.45	0.68±0.21	1.54±0.34	4.75±0.55	2.73±0.87	0.81±0.24	1.85±0.22	1.70±1.11
PGF2α	0.57±1.04	1.28±1.18	1.51±0.45	1.34±0.73	22.78±40.70	0.05±0.09	0.08±0.17	0.24±0.41	ND
TXB2	ND	0.32±0.24	1.31±0.61	1.01±0.81	37.94±81.32	ND	ND	0.20±0.33	ND
LTB_4	ND	62.68±33.18	1.44±0.48	2.53±0.59	5.94±1.32	ND	0.69±0.10	ND	0.44±0.15
5(6)-EET	7.45±4.26	127.06±81.24	430.53±125.27	159.34±79.07	4713±9624	32.27±16.17	27.48±29.87	94.50±189.72	45.72±5.57
14(15)-EET	22.20±1.10	30.74±3.33	100.06±26.09	35.49±18.83	1292±2718	6.74±4.37	2.11±1.69	18.68±36.52	10.06±4.50
5-HETE	0.03±0.07	17.41±7.51	16.98±6.24	13.90±7.97	525.04±1047.74	0.51±0.60	0.31±0.68	2.32±5.08	0.95±0.76
11-HETE	0.50±0.38	13.59±4.45	8.68±2.14	8.71±5.76	431.68±885.44	0.54±0.45	0.45±0.28	1.50±2.18	2.10±0.95

12-HETE	31±26	1326±427	747±481	352±233	8282±15408	10±7	37±56	82±84	42±24
15-HETE	11.06±6.22	151.24±53.90	679.04±187.37	173.74±96.90	6558±13996	44.86±35.59	10.71±9.08	140.9±281.4	63.86±39.64
5S,15S-DiHETE	0.01±0.03	49.61±15.68	51.29±13.93	43.59±28.08	780.93±1580.24	1.15±2.31	0.52±0.64	5.02±11.07	0.28±0.40
5,6-DHET	ND	9.44±1.63	2.12±1.20	4.41±4.33	35.85±45.41	0.14±0.13	0.94±1.32	2.71±4.57	2.02±1.04
8,9-DHET	0.25±0.36	7.44±1.56	5.26±2.40	6.17±6.20	70.13±96.90	1.11±0.95	0.83±1.47	3.22±6.17	2.74±0.63
11,12-DHET	0.39±0.15	19.76±2.84	12.77±5.15	16.67±18.57	136.38±210.45	2.35±1.57	2.59±2.83	9.31±16.58	7.62±3.01
14,15-DHET	17.40±4.12	454.05±73.56	328.07±131.31	403.37±508.51	2667.6±4226.5	77.19±43.93	75.14±70.35	226.6±388.4	241.3±69.8
5S,6S-LXA4	6.18±11.4	4.32±2.07	1.21±0.20	12.62±23.53	35.80±56.38	2.88±4.32	2.36±2.87	1.55±1.98	1.16±1.59
5S,6R-LXA4	2.10±4.62	1.34±0.53	0.51±0.45	4.75±8.28	21.67±36.04	2.22±4.61	9.40±15.72	2.13±2.65	0.06±0.08
15(R)-LXA4	5.18±11.45	4.99±9.10	1.48±0.97	4.86±6.19	182.51±394.55	0.31±0.70	7.96±16.80	0.95±2.13	0.04±0.06
DHA Metabolome									
DHA	864±91	10802±2817	6231±1817	12863±1689	44340±7078	145±58	619±73	857±153	157±115
4-HDoHE	0.19±0.02	3.63±1.07	1.15±0.12	3.37±0.47	167.07±331.25	0.24±0.04	1.80±0.32	0.20±0.03	0.31±0.03

7-HDoHE	1.67±0.34	12.06±3.45	3.88±1.41	7.62±6.71	289.82±55.61	0.57±0.07	1.03±0.16	0.66±0.10	0.08±0.01
13-HDoHE	0.76±0.17	15.00±7.00	2.87±0.78	5.03±4.31	191.73±0.37	0.19±0.04	0.20±0.04	0.32±0.04	0.44±0.06
14S-HDoHE	88.3±182.8	849.6±330.2	205.7±160.0	115.1±81.6	1850.7±3422.7	0.9±0.7	2.7±0.3	2.1±2.0	3.0±0.2
16-HDoHE	0.61±0.11	60.88±24.70	14.07±9.98	9.28±6.42	365.93±72.91	0	0.01±0.00	1.42±2.15	0.24±0.34
17-HDoHE	10.02±7.38	302.97±81.23	104.56±20.31	120.82±1141.86	1413.4±3001.9	4.16±2.67	7.24±2.02	23.61±45.20	6.93±4.64
20-HDoHE	7.92±8.61	136.21±46.14	140.43±22.88	79.64±46.08	1682.64±360.93	6.28±4.92	7.30±6.07	25.90±51.87	7.38±5.12
10S,17S-DiHDoHE	5.67±12.51	0.74±0.66	1.79±2.14	0.66±0.81	66.65±13.57	0.23±0.05	0.76±0.67	0.22±0.34	0
PD1 (NPD1)	0.11±0.02	0.72±1.03	7.22±2.18	0.93±0.63	3.19±8.49	1.12±1.03	0.55±1.02	2.01±4.36	0.30±0.05
17R-RvD1	0.04±0.01	0.30±0.45	0.14±0.16	0.10±0.14	4.41±5.47	0.14±0.14	0.17±0.22	0.01±0.01	0.05±0.05
RvD2	0.83±0.78	1.76±0.86	0.93±0.45	1.74±2.43	10.67±14.95	4.76±7.16	2.16±3.47	0.67±0.24	0.82±0.02
RvD3	1.79±1.78	27.21±8.30	7.60±5.51	31.24±17.91	552.24±100.98	17.06±10.79	29.92±29.57	7.03±1.96	10.32±11.17
RvD5	1.14±2.46	1.92±0.52	1.59±0.87	1.46±1.41	127.10±238.08	0.87±1.11	3.83±6.45	0.63±0.59	0.51±0.73
MaR1	1.82±3.77	17.75±5.14	4.86±5.07	9.39±17.72	556.60±1083.79	2.01±3.63	6.60±6.91	0.28±0.43	1.54±2.17

EPA Metabolome									
EPA	1.89±2.46	74.46±18.67	69.56±15.08	71.18±57.32	439.78±823.91	2.53±1.92	8.67±10.65	9.71±17.31	2.65±1.59
5-HEPE	0.13±0.29	1.97±0.79	1.91±1.07	2.26±1.74	67.53±117.01	0.57±0.62	0.92±1.23	0.49±0.63	0.69±0.98
8S-HEPE	0.85±1.35	3.85±4.69	29.41±10.74	11.31±8.77	292.46±598.57	2.50±3.64	5.23±6.50	4.98±9.34	4.96±7.01
9S-HEPE	0.12±0.27	10.75±2.99	46.61±14.98	15.29±9.09	457.08±960.10	2.85±2.23	1.53±1.06	9.97±21.44	3.70±2.30
12-HEPE	0.37±0.37	31.55±10.38	18.95±11.07	9.88±6.39	222.21±411.09	1.02±2.04	1.11±1.81	1.71±2.47	0.75±1.06
15-HEPE	50.5±20.2	725.9±286.7	2004.4±637.8	614.3±432.0	21447±45536	160.4±84.6	145.5±149.9	383.3±741.8	163.3±3.0
18-HEPE	13.01±8.15	17.87±9.43	48.55±13.72	16.90±12.28	414.28±755.94	34.43±39.16	36.03±48.59	10.09±15.70	16.67±10.65
RvE1	6.07±4.71	18.70±17.97	15.10±3.11	19.74±11.84	142.29±262.87	17.72±5.96	27.59±38.33	6.84±3.19	4.33±0.49
PGD3	1.54±1.88	1.22±0.65	2.32±0.90	1.07±1.10	19.53±36.28	2.33±3.89	2.07±1.43	1.83±1.76	50.92±69.76
DPA Metabolome									
DPA	44.1±45.9	336.0±119.2	277.5±104.7	582.3±842.6	1569.7±2464.1	12.0±5.8	34.6±53.2	37.3±65.6	9.0±4.6
17-ketoDPA	2.53±3.06	63.13±12.36	13.72±3.08	22.36±29.42	112.90±202.80	1.70±0.87	5.61±5.63	4.38±5.87	3.80±4.62
RvDp5	3.42±0.86	8.23±1.69	28.92±9.79	8.93±0.76	7.97±0.32	0.99±0.45	0.13±0.03	4.00±0.53	0.21±0.08
